# Reference Estimates of Mean Tear Film Lipid Layer Thickness Measured with LipiView^®^ and LipiView II^®^ in Healthy Adults: A Systematic Review and Meta-Analysis

**DOI:** 10.3390/jcm15176826

**Published:** 2026-09-03

**Authors:** Maria Sobol, Jacek Pniewski

**Affiliations:** 1 Department of Biophysics, Physiology and Pathophysiology, Medical University of Warsaw, Chałubińskiego 5, 02-004 Warszawa, Poland; 2 Faculty of Physics, University of Warsaw, Pasteura 5, 02-093 Warszawa, Poland; j.pniewski@uw.edu.pl

**Keywords:** tear film lipid layer thickness, LipiView, interferometry, ocular surface, reference estimates, healthy adults, systematic review, meta-analysis

## Abstract

**Background/Objectives**: Tear film lipid layer thickness (LLT) is an important parameter of ocular surface function and can be objectively measured using LipiView ocular surface interferometry. However, reference LLT estimates in clinically healthy populations remain inconsistent. This systematic review and meta-analysis aimed to estimate pooled reference LLT values and explore potential sources of variability. **Methods**: A systematic search of PubMed, Scopus, and Web of Science was performed according to PRISMA 2020 guidelines. Studies reporting quantitative LLT measurements obtained using LipiView in healthy or clinically screened adults were included. Primary and expanded meta-analyses were conducted using random-effects models, with a fixed-effect model additionally applied as a sensitivity analysis for the primary meta-analysis. Subgroup analyses evaluated the influence of contact-lens use history, age group, and population eligibility criteria. **Results**: Twelve studies including 451 participants were included. The primary meta-analysis of three reference cohorts (88 participants) showed a pooled mean LLT of 62.59 nm (95% CI: 59.14–66.04 nm). The expanded analysis yielded a comparable pooled mean LLT of 64.41 nm (95% CI: 57.50–71.31 nm), with substantial heterogeneity (I^2^ = 98.71%). Sensitivity analysis in the expanded meta-analysis demonstrated that the pooled estimate was not substantially influenced by any individual study. Exploratory subgroup analyses indicated numerically lower pooled LLT in studies with documented contact-lens exposure and higher pooled LLT in studies with older populations. However, none of the between-subgroup differences reached statistical significance, and these findings should be interpreted cautiously. **Conclusions**: A pooled mean LLT of approximately 62–64 nm may serve as a reference estimate for clinical interpretation rather than a diagnostic threshold. However, given the substantial clinical and methodological heterogeneity among available studies, this estimate should be interpreted as a descriptive summary of LipiView-derived LLT values rather than a precise biological parameter. Further standardized studies are needed to establish reliable normative values and clinically meaningful diagnostic criteria.

## 1. Introduction

The precorneal tear film is a complex structure vital for maintaining ocular surface integrity, providing immunological protection, and ensuring a smooth optical surface for clear vision [1,2]. It is traditionally described as a trilaminar or multi-layered structure consisting of a mucin phase, an aqueous phase, and an outermost surface tear film lipid layer (TFLL). Produced primarily by the Meibomian glands located in the tarsal plates, the TFLL typically ranges in thickness from 40 to 100 nm in healthy individuals. This thin layer performs critical homeostatic functions, including stabilizing the tear film by lowering surface tension and acting as a barrier to prevent excessive evaporation of the underlying aqueous phase [3,4,5].

These physiological functions are clinically relevant because dry eye disease (DED) and meibomian gland dysfunction (MGD) are common conditions. The TFOS DEWS III Digest reported that DED prevalence estimates based on the TFOS DEWS II diagnostic criteria ranged from 5.4% to 44.2% across the populations studied, with substantial variation according to age, sex, population characteristics, and diagnostic approach [4]. A Bayesian synthesis estimated the overall global prevalence of DED at 11.59%, although the estimate increased to 29.5% when the analysis was restricted to studies applying the TFOS DEWS II diagnostic criteria [6]. MGD, a major contributor to evaporative DED, has an estimated pooled global prevalence of 35.8% [7]. The high prevalence of these conditions, together with the substantial variation associated with diagnostic definitions and population characteristics, highlights both the public-health relevance of tear film dysfunction and the need for objective parameters with appropriately characterized reference values. In this context, LLT may provide useful information on tear film and meibomian gland status, although it should not be interpreted as a stand-alone diagnostic test.

In clinical practice, tear film lipid layer thickness (LLT) has emerged as a fundamental objective biomarker for evaluating the ocular surface, particularly in the context of meibomian gland dysfunction (MGD) and evaporative dry eye disease (DED). A reduction in LLT is often associated with Meibomian gland loss and tear film instability [8]. Some studies have applied thresholds, such as LLT < 60 nm or <70 nm, to identify patients at higher risk for significant dry eye symptoms [9,10,11,12,13,14,15].

Despite its clinical importance, establishing a definitive “normal” range for LLT in healthy populations remains challenging due to the high degree of variability reported across existing literature. Mean LLT values for healthy controls vary significantly between cohorts, with some studies reporting averages near 50–60 nm [16,17], while others report values exceeding 75–80 nm [15,18]. This heterogeneity may be attributed to various factors, including demographic differences such as age and sex, blinking patterns (where incomplete blinks may impair the distribution of the tear film lipid layer), temperature, humidity, medication use, lifestyle-related factors, ocular surface disorders such as MGD, and environmental stressors like air pollution. Furthermore, while some research suggests LLT remains relatively constant throughout the day, other data indicate potential diurnal variations or shifts related to environmental factors like prolonged mask use [19,20,21,22,23]. In addition, the obtained values may depend on the selected region of interest and the method used to average measurements [24].

A secondary challenge in defining normal LLT lies in the lack of standardization among diagnostic platforms. Since 1998, several commercial instruments—including the Tearscope/Tearscope Plus (Keeler Ltd., Windsor, Berkshire, UK), EasyTear^®^ VIEW+ (SBM Sistemi, Orbassano, Italy), and Keratograph 5M (Oculus GmbH, Wetzlar, Germany)—have enabled qualitative assessment of the tear film lipid layer based on its interference patterns. These patterns are commonly classified according to the Guillon grading system and related to approximate ranges of lipid layer thickness [24,25,26].

Currently, LLT is measured using various interferometric systems, such as the LipiView^®^/LipiView II^®^ (TearScience, Inc., Morrisville, NC, USA; currently Johnson & Johnson Vision, Jacksonville, FL, USA) and IDRA^®^ Ocular Surface Analyzer (SBM Sistemi, Torino, Italy). A limited number of studies have indicated that these platforms are not fully interchangeable, as they utilize different measurement principles and display significant inter-instrument variability [17,27,28]. Comparability between studies is further restricted by device-specific features, including proprietary processing algorithms and upper measurement or reporting limits, commonly set at 100 nm.

Despite the increasing use of automated interferometry in both clinical practice and research, no comprehensive synthesis of LLT measurements in healthy individuals has been available. Consequently, clinicians and investigators lack an evidence-based reference benchmark against which individual measurements and study findings can be interpreted. This systematic review and meta-analysis were therefore undertaken to synthesize the available evidence and estimate pooled reference LLT values in healthy populations. Providing these benchmarks may facilitate interpretation of LLT measurements, improve comparability across studies, and support future investigations of ocular surface disease and treatment outcomes.

## 2. Materials and Methods

### 2.1. Search Strategy and Study Selection

This systematic review and meta-analysis was conducted in accordance with the Preferred Reporting Items for Systematic Reviews and Meta-Analyses (PRISMA) 2020 guidelines [29,30]. A systematic literature search was performed in the PubMed, Scopus, and Web of Science databases to identify eligible studies. The search included all records available from database inception to 23 August 2026, without restrictions on publication date, and was limited to articles published in English.

The initial search strategy was intentionally focused on the outcome of interest, lipid layer thickness (LLT), and its measurement using the LipiView, using the expression “lipid layer thickness” AND “LipiView” AND “eye”. Following revision, an additional broader search was performed using the terms “lipid layer thickness”, “LLT”, “tear film lipid layer”, “LipiView”, “LipiView II”, “interferometry”, and “ocular surface interferometer”. Records identified through the initial and expanded search strategies were combined and deduplicated before screening. The final updated search was completed on 23 August 2026. Complete database-specific search strategies, including search fields, applied limits, and search dates, are provided in Supplementary Table S1a,b.

**Figure 1:**
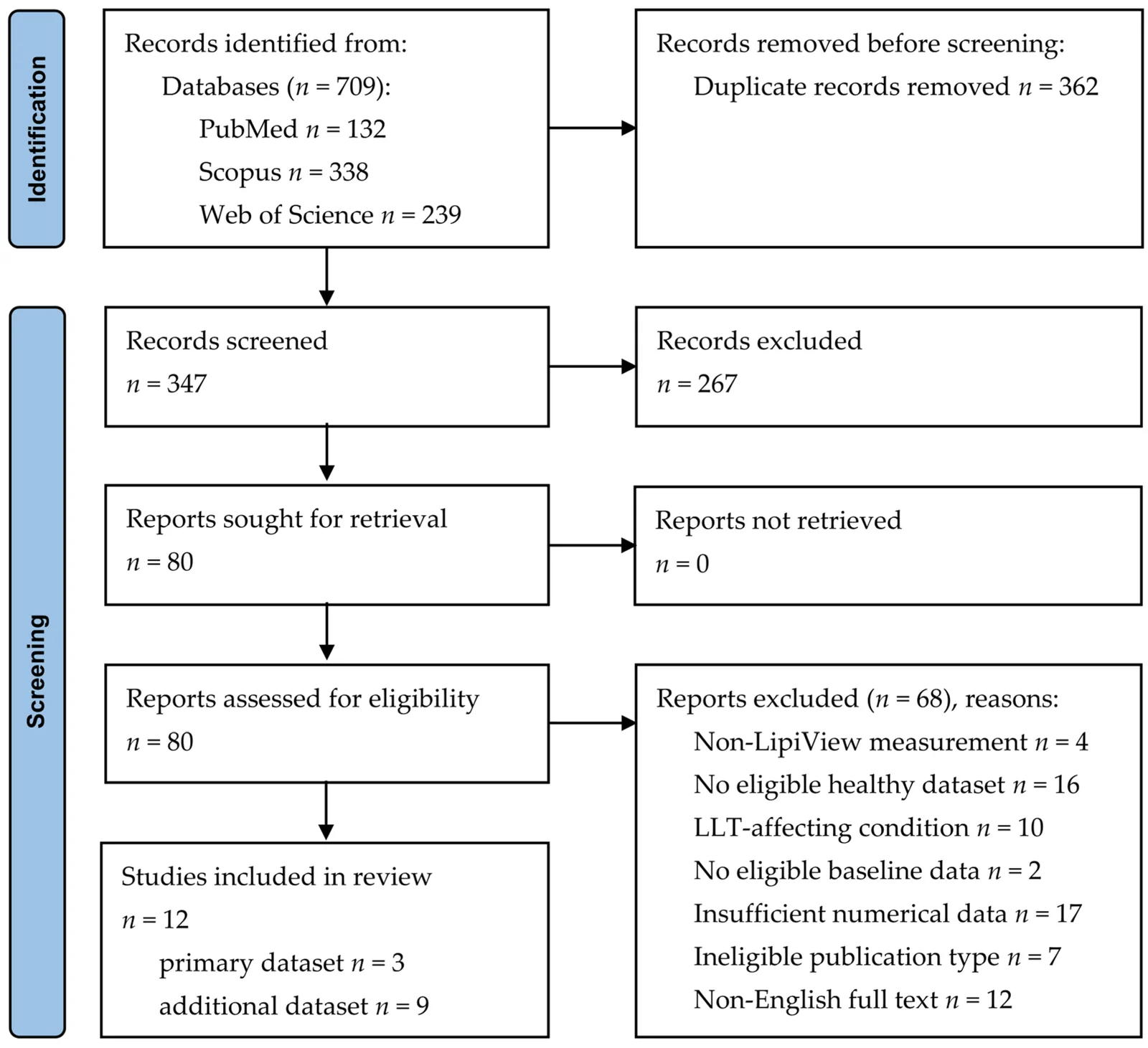
Flow chart for inclusion of studies. Only exclusion criteria with a non-zero number of publications are listed.

The literature search, study selection, data extraction, and data verification were performed manually by the investigators. Two reviewers (MS and JP) independently screened titles, abstracts, and full-text articles for eligibility according to predefined inclusion and exclusion criteria. Any discrepancies were resolved through discussion and critical re-evaluation until consensus was achieved. As full agreement was reached for all decisions, consultation with a third reviewer was not required. The flow chart of the process is shown in Figure 1.

The review was not prospectively registered, and no formal review protocol was prepared. Nevertheless, the research question, eligibility criteria, target measurement method (LipiView/LipiView II), and objective of estimating LLT in clinically healthy or clinically screened adult populations were defined before study screening. In contrast, the distinction between the primary and expanded analytical cohorts was developed during evaluation of the eligible literature to account for differences in the degree of clinical characterization of the included populations. Sensitivity analyses and exploratory subgroup analyses were likewise undertaken after review of the available study characteristics and should therefore be considered post hoc or exploratory rather than prospectively prespecified analyses.

### 2.2. Review Question and Adapted PICO Framework

The review question was: “What are the mean LipiView-derived tear film lipid layer thickness values in healthy or clinically screened adults without clinically significant ocular surface disease?” Because this review evaluated a quantitative measurement rather than a therapeutic intervention, eligibility criteria were structured using an adapted PICO framework in which the intervention component was interpreted as the index measurement:

Population: adults aged ≥ 18 years who were clinically healthy, asymptomatic, included as healthy controls, or clinically screened and free from clinically significant ocular surface disease at the time of LLT assessment.

Intervention/index measurement: quantitative LLT assessment performed using the LipiView or LipiView II ocular surface interferometer.

Comparison: no comparator group was required for inclusion; where sufficient data were available, comparisons were performed between the primary reference cohort and the additional studies included only in the expanded analysis, and between subgroups defined by reported contact-lens exposure and age.

Outcome: mean LLT expressed in nanometers, together with a measure of variability sufficient for quantitative synthesis, such as the standard deviation or equivalent statistics.

### 2.3. Eligibility Criteria

Eligibility was assessed at the study, participant, measurement, and outcome levels. General eligibility criteria were first applied to determine whether a study contained potentially relevant data. Eligible datasets were then assigned hierarchically to the primary or expanded quantitative synthesis according to the characteristics and recruitment context of the analyzed population.

#### 2.3.1. General Inclusion Criteria

Studies were eligible for consideration if they met all of the following criteria:

1. They were original, peer-reviewed human studies employing a prospective, cross-sectional, randomized, or other observational design.
2. Participants were adults aged ≥ 18 years. Studies involving mixed-age populations were eligible only when data for adults could be extracted separately.
3. Quantitative tear film lipid layer thickness was measured using the LipiView or LipiView II ocular surface interferometer.
4. The study contained an identifiable cohort or subgroup without clinically significant ocular surface disease at the time of LLT assessment.
5. Mean LLT and a corresponding standard deviation were reported, or sufficient numerical data were available to derive the mean and its variability.
6. The full-text article was published in English.

If a study included several participant groups, only data from groups meeting the eligibility criteria were extracted.

#### 2.3.2. Hierarchical Population Criteria for the Primary and Expanded Meta-Analyses

Eligible datasets were classified hierarchically according to the characteristics and recruitment context of the study population.

Primary meta-analysis. The primary meta-analysis applied the more restrictive criteria and included datasets from studies explicitly enrolling clinically healthy or asymptomatic adults. Participants were required to have a well-characterized ocular surface status and no clinically significant ocular surface disease at the time of LLT assessment. These datasets constituted the primary reference cohort.

Expanded meta-analysis. The expanded meta-analysis included all datasets contributing to the primary meta-analysis and additionally incorporated eligible datasets from:

1. Healthy control groups embedded in studies of ocular surface disease, treatment, or other clinical conditions;
2. Clinically screened populations, such as preoperative refractive surgery candidates, provided that LLT was measured before the procedure and no clinically significant ocular surface disease was present at the time of assessment;
3. Healthy control groups or otherwise eligible clinically screened participants recruited in exposure-based studies.

These additional datasets constituted the expanded-reference component. Together with the primary reference cohort, they formed the complete dataset used in the expanded meta-analysis. This nested, hierarchical approach was used to obtain a more restrictive reference estimate in the primary analysis and to evaluate the effect of broader population eligibility on the pooled estimate in the expanded analysis.

Previous contact-lens wear was not considered an automatic exclusion criterion, provided that participants had no clinically significant ocular surface disease at the time of measurement. Contact-lens history was recorded when reported and subsequently evaluated in a subgroup analysis.

#### 2.3.3. Exclusion Criteria

Studies or datasets were excluded if they met any of the following criteria:

1. LLT was measured using a method or device other than LipiView or LipiView II, including qualitative Guillon grading, high-resolution microscopy, absolute-reflectance methods, or the IDRA ocular surface analyzer.
2. The study included exclusively pediatric participants or did not provide separately extractable data for adults.
3. No clinically healthy, asymptomatic, healthy-control, or otherwise eligible clinically screened dataset could be extracted separately.
4. Participants had dry eye disease, meibomian gland dysfunction, nasolacrimal duct obstruction, rosacea, thyroid eye disease, neurotrophic keratitis, or another ocular or systemic condition considered likely to affect LLT.
5. The purported reference group consisted solely of fellow eyes of participants with unilateral ocular disease.
6. Only post-treatment, postoperative, or post-exposure LLT measurements were available, without eligible baseline or untreated control data.
7. The study did not report sufficient numerical information to calculate mean LLT and an associated measure of variability.
8. The publication was a review, editorial, letter, conference abstract, case report, protocol, or another article without original eligible LLT data.
9. The study involved animals, ex vivo material, or in vitro experiments.
10. The publication duplicated a dataset reported in another included article; in such cases, the most complete eligible dataset was retained.
11. The full-text article was not published in English.

Authors were not contacted to obtain missing or additional numerical data.

### 2.4. Statistical Analysis

Statistical analyses were performed using TIBCO Software Inc. Statistica, version 13 (TIBCO Software Inc., Palo Alto, CA, USA). Pooled mean LLT values were calculated with corresponding 95% confidence intervals (CIs). Study-level estimates were pooled using inverse-variance weighting. For random-effects models, between-study variance (τ^2^) was estimated using the DerSimonian–Laird method. Two-sided 95% confidence intervals for pooled estimates were calculated using the normal approximation (z-based method). As an additional sensitivity analysis, the random-effects models were re-estimated using restricted maximum likelihood (REML) for between-study variance and the Hartung–Knapp adjustment for the confidence intervals of the pooled estimates. Statistical heterogeneity among studies was assessed using Cochran’s Q test and quantified using the I^2^ statistic, with values of 25–50%, 50–75%, and >75%, indicating low, moderate, and high heterogeneity, respectively.

Two quantitative syntheses were performed. The primary meta-analysis included studies of clinically healthy or asymptomatic participants with a well-characterized ocular surface status, whereas the expanded meta-analysis additionally included well-characterized healthy control groups and clinically screened populations. Each publication contributed one study-level estimate to each applicable analysis. Random-effects models were used as the primary analytical approach for both syntheses because clinical and methodological variability among study populations and measurement procedures was anticipated, irrespective of the observed statistical heterogeneity. A fixed-effect model was additionally applied as a sensitivity analysis for the primary meta-analysis. For the expanded analysis, a 95% prediction interval was additionally calculated to describe the expected range of true mean LLT values across comparable populations.

Forest plots were generated to present individual study-level estimates, pooled mean LLT values, corresponding 95% confidence intervals, and study weights. For the primary meta-analysis, the robustness of the pooled result to model choice was assessed by comparing the random-effects analysis with a fixed-effect sensitivity analysis. Both models yielded identical results because the estimated between-study variance was zero τ^2^ = 0. The robustness of the random-effects estimates was additionally assessed using REML with the Hartung–Knapp adjustment. Leave-one-out and publication-bias analyses were not performed for the primary meta-analysis because only three studies were included, making such assessments statistically unreliable and difficult to interpret. The expanded meta-analysis included 12 publications, each contributing one study-level estimate. A leave-one-out sensitivity analysis was therefore conducted by sequentially omitting each study and recalculating the pooled estimate to evaluate whether the overall result was disproportionately influenced by any individual study. When separate results for both eyes were reported, right-eye data were selected using a consistent, non-outcome-based rule to avoid including correlated estimates from the same participants. A sensitivity analysis was performed by replacing the right-eye estimates with the corresponding left-eye estimates while keeping all other study data unchanged.

Risk of bias was assessed using the Joanna Briggs Institute (JBI) Critical Appraisal Tool for Analytical Cross-sectional Studies, with disagreements resolved by consensus. Although the included publications originated from different study designs, only baseline or eligible healthy/control cross-sectional LLT estimates contributed to the meta-analysis; therefore, the JBI tool was applied to the specific dataset contributing to the pooled analysis rather than to the original study design as a whole. Overall risk of bias was classified using the following criteria. Studies were considered to have low risk of bias when all applicable domains were rated “yes.” Studies with one or more domains rated “unclear,” but no critical domain rated “no,” were classified as having low-to-moderate risk of bias. Studies with a “no” judgment in at least one critical domain were classified as having high risk of bias. These overall categories were study-defined summary classifications used to facilitate presentation and are not standard JBI categories. They were not derived from a numerical summary score. The results of the risk-of-bias assessment are presented in Table 1 and Supplementary Table S2.

Potential small-study effects and publication bias were explored through visual inspection of the funnel plot, Egger’s regression test, the Begg–Mazumdar rank correlation test, and the trim-and-fill method. The trim-and-fill procedure was used to assess how potentially missing studies might influence the pooled mean LLT estimate. Given the relatively small number of included studies (*n* = 12) and the considerable between-study heterogeneity, all analyses of funnel-plot asymmetry and small-study effects were considered exploratory and interpreted cautiously.

Subgroup analyses were performed to evaluate the influence of study population characteristics on LLT estimates. First, an exploratory subgroup analysis according to population eligibility compared the primary reference cohort with the expanded-reference component, defined as the additional studies included only in the expanded meta-analysis. Within the expanded meta-analysis, an additional subgroup analysis compared studies with documented contact-lens exposure and studies explicitly reporting no contact-lens exposure. The overall “Summary” estimates displayed in the subgroup forest plots were calculated by combining the subgroup-specific pooled estimates and may therefore differ from the pooled estimate obtained directly from all individual study-level estimates in the expanded meta-analysis. These subgroup-level summary values were presented for completeness and were not interpreted as alternative overall estimates of LLT. The overall pooled estimate for the expanded meta-analysis was calculated directly from all 12 study-level estimates.

Because individual-level data were not consistently available, the effective sample size was based on the number of participants whenever possible, according to the approach reported in each original study.

## 3. Results

The search strategy identified 132 articles in PubMed, 337 in Scopus, and 239 in the Web of Science databases. After screening the records identified using the search strategy, twelve studies were included: Dutta et al., 2019 [31], Stegmann et al., 2019 [32], Kengpunpanich et al. 2025 [33], Kumar et al., 2025 [16], Kim et al., 2022 [15], Ji et al., 2022 [34], Singh et al., 2023 [17], Al Sabti et al., 2022 [11], Carreira et al., 2022 [10], Markoulli et al., 2018 [35], Eom et al., 2013 [36], and Fukuoka et al., 2017 [37]. Table 2 summarizes the demographic characteristics of the participants and the methodological characteristics of the included studies, such as age, sex, number of subjects, eye selection for analysis, history of contact-lens use (when reported) and LLT with SD. The complete study-level dataset used for the quantitative analyses, including the selected estimates, standard errors, and key analytical selections, is provided in Supplementary Table S3.

### 3.1. Primary Meta-Analysis

In the primary meta-analysis, a core dataset of strictly selected reference cohort studies was evaluated, consisting of three studies: Dutta et al., 2019 [31], Stegmann et al., 2019 [32], and Kengpunpanich et al., 2025 [33]. These publications were selected based on strict inclusion criteria to ensure methodological consistency and a well-defined participant population representative of a reference (near-healthy) ocular surface cohort. Across the included studies, participants were characterized by standardized ophthalmic assessment and minimal presence of confounding ocular surface disease, resulting in a relatively homogeneous sample and reduced selection bias. This approach was intended to improve internal validity and provide a more stable estimate of the pooled effect. In total, 88 subjects were included in the analysis, with a predominance of female participants (*n* = 58, 65.9%) compared with male participants (*n* = 30, 34.1%).

**Figure 2:**
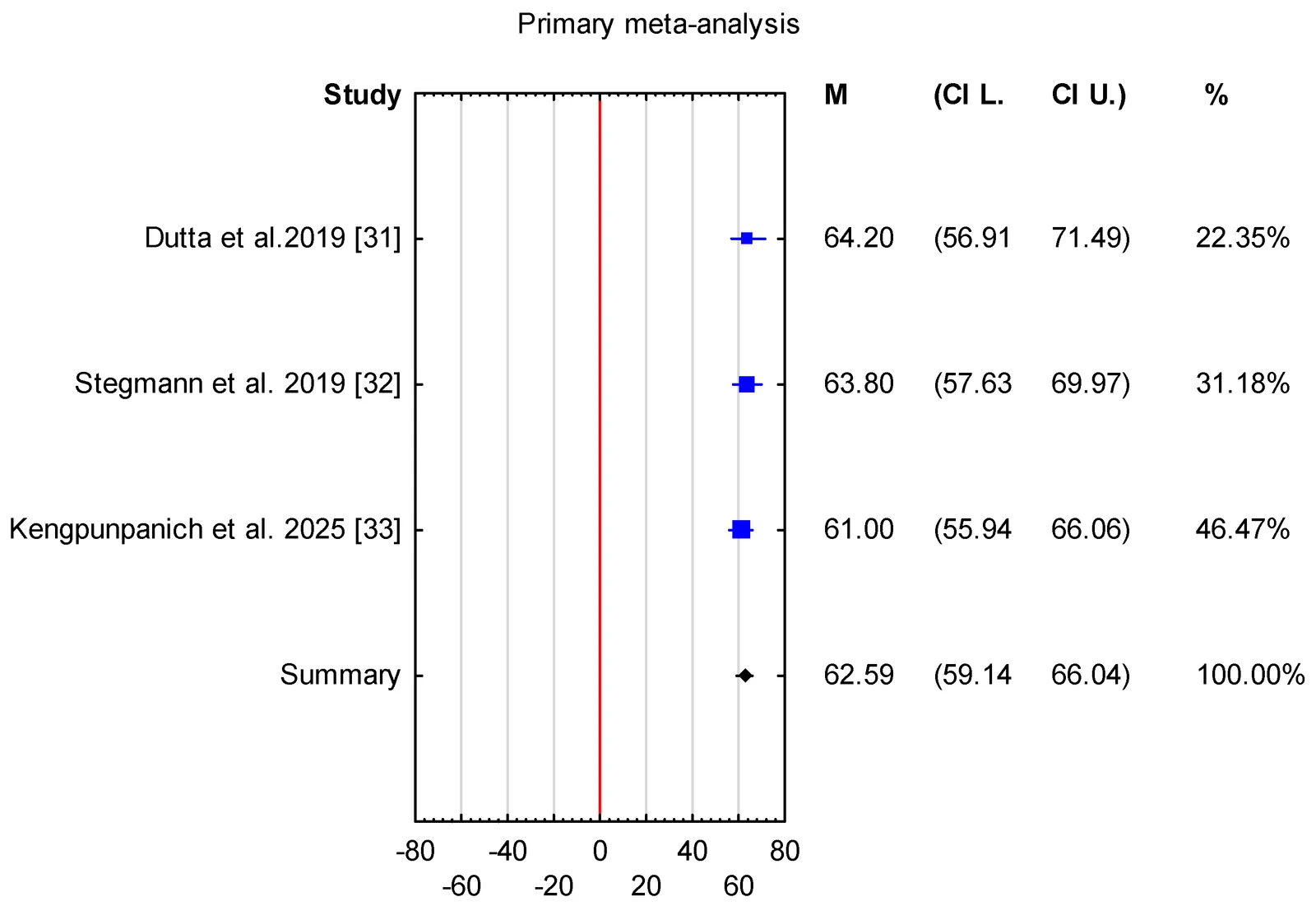
Forest plot of the mean LLT [nm]. Blue squares represent individual study point estimates, with square size proportional to the study weight in the pooled estimate. Horizontal lines indicate 95% CIs. The black diamond represents the pooled estimate.

The sample sizes of the individual studies ranged from 10 to 60 participants. For all studies, the sample size used in the meta-analysis corresponded to the number of participants rather than the number of eyes. Dutta et al. [31] included data from both eyes; however, measurements from both eyes were averaged before the final analysis. Kengpunpanich et al. [33] also included data from both eyes, but the statistical analysis accounted for the inter-eye correlation using generalized estimating equations (GEE). Accordingly, the effective sample sizes for these studies were based on the number of participants (18 and 60, respectively). Stegmann et al. [32] analyzed one eye per participant (10 eyes from 10 individuals). Kengpunpanich et al. [33] reported results for both the entire cohort and separate age groups. To ensure that each publication contributed only one independent study-level estimate and to avoid disproportionately weighting this study, the overall results for the entire cohort were included in the revised meta-analysis instead of the age-stratified estimates. Thus, the primary meta-analysis comprised three publications, each contributing one study-level estimate. Using a random-effects model, the pooled mean LLT was 62.59 nm (95% CI: 59.14–66.04 nm) (Figure 2). Statistical heterogeneity was not detected: Q = 0.745, df = 2, *p* = 0.689, I^2^ = 0.00% (95% CI: 0.00–90.99%), τ^2^ = 0.00 nm^2^ (95% CI: 0.00–95.70 nm^2^). Nevertheless, the wide confidence intervals around the heterogeneity estimates indicate considerable uncertainty due to the small number of included studies. The fixed-effect sensitivity analysis yielded the same pooled estimate and confidence interval because the estimated between-study variance was zero. Therefore, a separate fixed-effect forest plot is not presented, as it would duplicate the random-effects forest plot. In an additional sensitivity analysis using REML with the Hartung–Knapp adjustment, the pooled mean LLT was 62.59 nm (95% CI: 58.07–67.11 nm; τ^2^ = 0.00 nm^2^). The point estimate was unchanged, although the confidence interval was wider, reflecting the greater uncertainty associated with the small number of included studies.

### 3.2. Expanded Meta-Analysis

**Figure 3:**
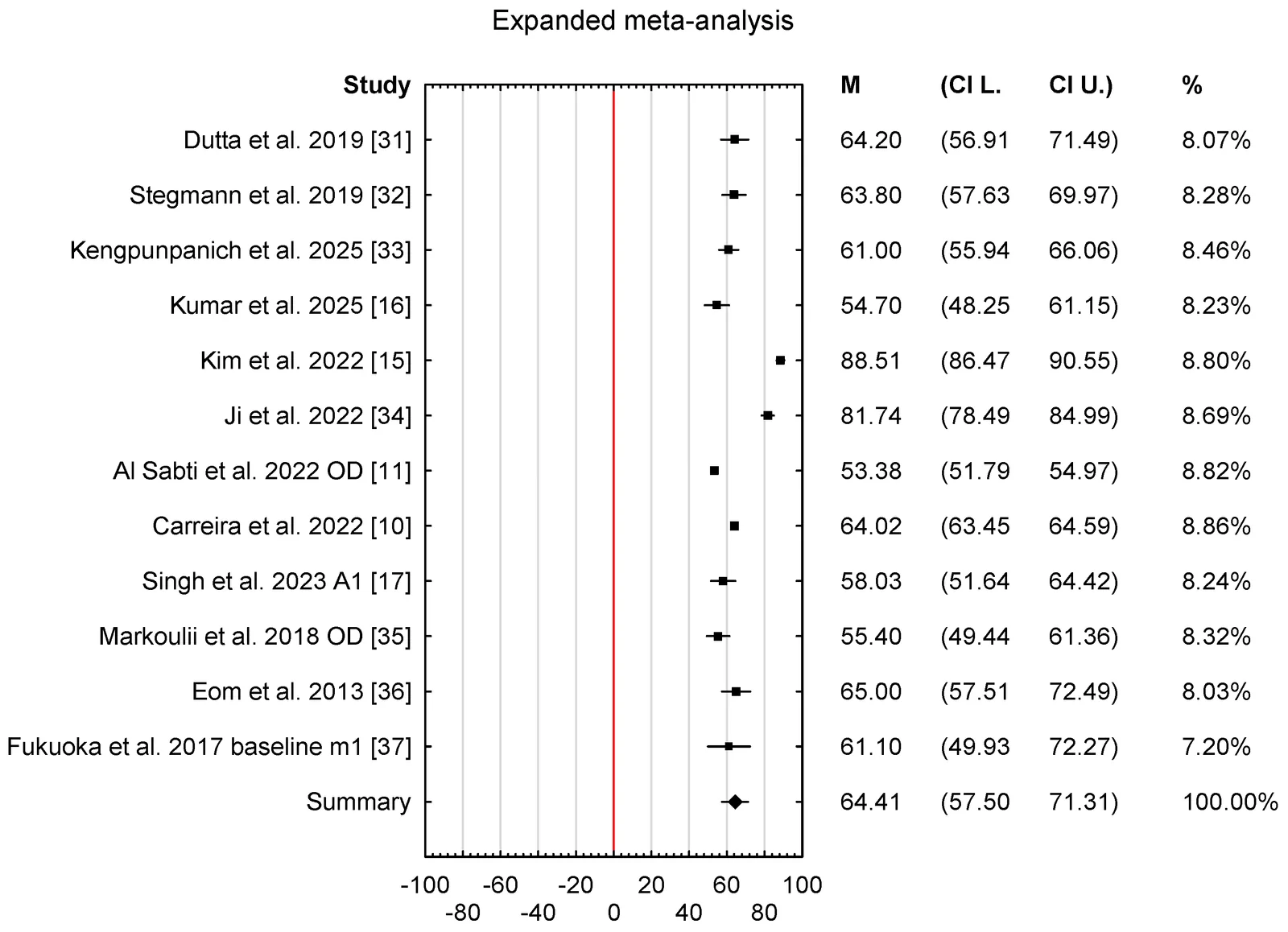
Forest plot of the mean LLT [nm] in the expanded participant group. Squares represent the point estimates for individual studies, with their size proportional to the study weight in the pooled estimate. Horizontal lines indicate the corresponding 95% confidence intervals (95% CIs). The diamond represents the pooled mean estimate and its 95% CI.

To evaluate the influence of broader study populations on the pooled estimate, an expanded meta-analysis was performed by incorporating an additional nine studies comprising well-characterized healthy control groups or clinically screened populations that were not originally recruited to establish reference cohorts. Each publication contributed a single study-level estimate to the analysis. This analysis included a total of 12 studies involving 451 participants (Figure 3).

In the studies by Kumar et al. [16], Kim et al. [15], Ji et al. [34], and Carreira et al. [10], one eye per participant was included in the analysis. Al Sabti et al. [11] and Markoulli et al. [35] reported separate results for the right and left eyes. Therefore, right-eye estimates were used in the main expanded analysis according to a consistent, non-outcome-based selection rule. In the sensitivity analysis, replacing these estimates with the corresponding left-eye results yielded a pooled mean LLT of 64.62 nm (95% CI: 57.58–71.67 nm) shown in Supplementary Figure S1, compared with 64.41 nm (95% CI: 57.50–71.31 nm) in the expanded analysis. Heterogeneity remained comparable (I^2^ = 98.79%, τ^2^ = 145.87 nm^2^), indicating that the choice of eye did not materially affect the pooled estimate. Fukuoka et al. [37] reported two pre-intervention LLT measurements obtained on separate visits. The first-visit baseline value (61.10 ± 39.10 nm) was used in the main analysis. In a sensitivity analysis, this estimate was replaced with the preinstillation value measured immediately before diquafosol administration (62.30 ± 31.10 nm), yielding a pooled mean LLT of 64.48 nm (95% CI: 57.61–71.36 nm). Thus, the choice between the two pre-intervention measurements did not materially affect the pooled estimate. Singh et al. [17] reported repeated and observer-specific LipiView measurements. To avoid including multiple correlated estimates from the same publication, a single study-level estimate (Observer 1, measurement II: 58.03 ± 17.85 nm) was retained in the main expanded analysis. As no single repeated measurement was methodologically preferable, Observer 1, measurement II was selected as a single representative study-level estimate independently of its value, and alternative reasonable choices were examined in sensitivity analyses. Replacing the selected estimate with Observer 1, measurement I (59.70 ± 17.21 nm) yielded a pooled mean LLT of 64.54 nm (95% CI: 57.65–71.44 nm), whereas use of Observer 2, measurement I (56.70 ± 16.47 nm) yielded a pooled mean LLT of 64.29 nm (95% CI: 57.39–71.19 nm). Between-study heterogeneity remained essentially unchanged (I^2^ approximately 98.71–98.79%), indicating that the selection of observer-specific measurement did not materially influence the pooled result. Stegmann et al. [32] analyzed one eye per participant (10 eyes from 10 individuals). Dutta et al. [31] collected measurements from both eyes, but the mean LLT value across both eyes was used for each participant in the final analysis. Similarly, Kengpunpanich et al. [33] included measurements from both eyes, but the statistical analysis accounted for the inter-eye correlation using generalized estimating equations (GEE); therefore, the sample size was based on the number of participants rather than the number of eyes.

The majority of participants were female. Participants ranged in age from 18 to 78 years, and the sample sizes of the individual studies ranged from 10 to 80 participants. Quantitative LLT measurements were obtained using the LipiView or LipiView II ocular surface interferometer in all included studies. Using a random-effects model, the pooled mean LLT was 64.41 nm (95% CI: 57.50–71.31 nm) (Figure 3). Considerable between-study heterogeneity was observed (Q = 851.6, df = 11, *p* < 0.001, I^2^ = 98.71%, 95% CI: 98.48–99.04%; τ^2^ = 139.4 nm^2^, 95% CI: 112.74–183.02 nm^2^). The 95% prediction interval ranged from 36.96 to 91.86 nm, indicating substantial variation in the true mean LLT expected across comparable populations and supporting cautious interpretation of the pooled estimate.

Among the individual studies, the highest mean LLT was reported by Kim et al. [15] (88.51 nm; 95% CI: 86.47–90.55 nm), whereas the lowest was observed for the right eye in Al Sabti et al. [11] OD (53.38 nm; 95% CI: 51.79–54.97 nm). The remaining studies reported mean values ranging from 54.70 to 81.74, with confidence intervals of varying width, reflecting differences in the precision of the individual estimates.

#### 3.2.1. Sensitivity Analysis

**Figure 4:**
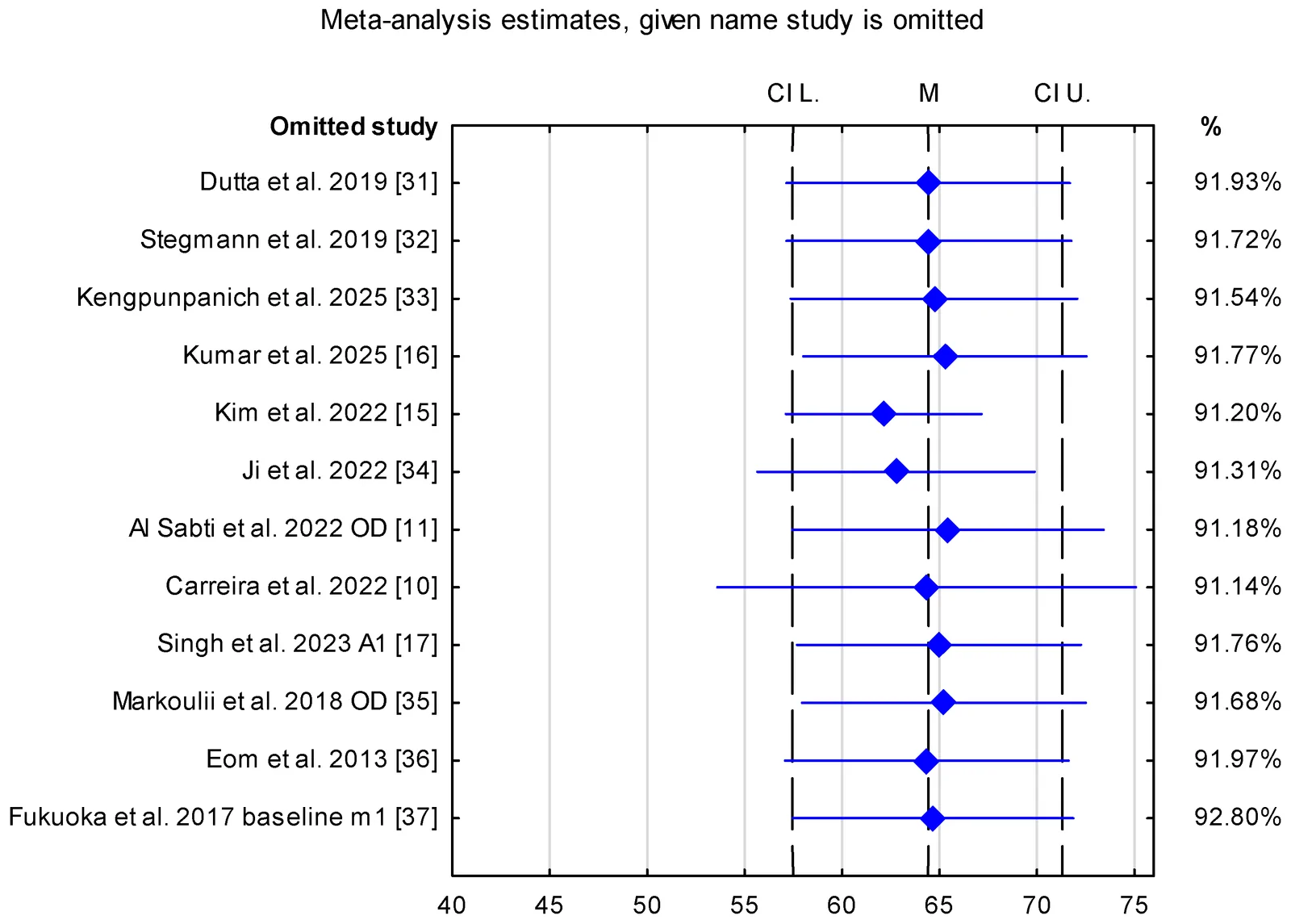
Sensitivity analysis of individual studies on the pooled mean LLT [nm] with 95% confidence intervals. Each study listed on the *Y*-axis was sequentially omitted to evaluate its impact on the overall pooled estimate. The percentage column represents the sum of the original meta-analytic weights of the studies remaining after omission of the indicated study (100% minus the original weight of the omitted study).

Leave-one-out sensitivity analysis confirmed the robustness of the pooled LLT estimate. Sequential omission of each individual study resulted in only negligible changes in the pooled mean estimate, with substantial overlap of the corresponding 95% confidence intervals across all iterations. No individual study exerted a disproportionate influence on the overall pooled estimate, indicating that the findings were robust and not driven by any single study (Figure 4).

An additional sensitivity analysis using REML with the Hartung–Knapp adjustment yielded a pooled mean LLT of 64.44 nm (95% CI: 57.53–71.35 nm; τ^2^ = 115.27 nm^2^), closely consistent with the main random-effects estimate and indicating that the overall interpretation was not materially affected by the alternative estimation approach.

#### 3.2.2. Publication Bias

**Figure 5:**
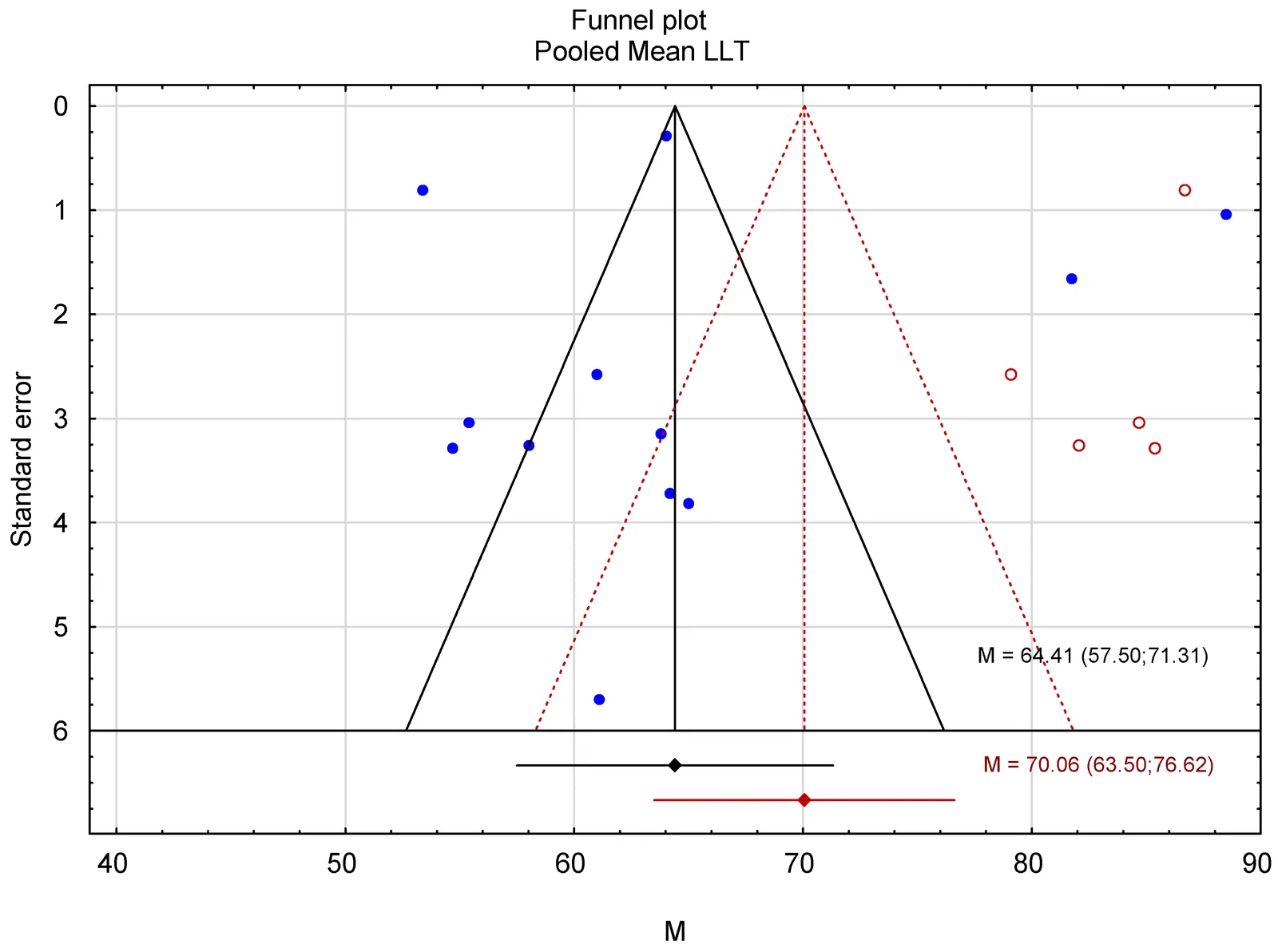
Funnel plot with trim-and-fill adjustment for the expanded meta-analysis. Filled blue squares represent observed studies and open red circles imputed studies. Solid black lines indicate the original estimate, and red dashed lines the trim-and-fill-adjusted estimate.

Potential small-study effects and publication bias were assessed by visual inspection of the funnel plot, the trim-and-fill method, Egger’s regression test, and the Begg–Mazumdar rank correlation test. Visual inspection suggested possible funnel-plot asymmetry, and the trim-and-fill procedure imputed three potentially missing studies on the right side of the plot. The adjusted pooled mean LLT increased from 64.41 nm (95% CI: 57.50–71.31 nm) to 70.06 nm (95% CI: 63.50–76.62 nm) (Figure 5). Although the adjusted estimate was higher than the original pooled estimate, the confidence intervals overlapped substantially.

Neither Egger’s regression test (intercept = 0.909, 95% CI: −6.742 to 8.560; t = 0.265, df = 10, *p* = 0.797) nor the Begg–Mazumdar rank correlation test (τb = −0.333, Z = −0.522, *p* = 0.602) provided statistically significant evidence of funnel-plot asymmetry. Nevertheless, the trim-and-fill findings and apparent visual asymmetry should be interpreted cautiously. Given the relatively small number of included studies (*n* = 12) and the considerable between-study heterogeneity, these analyses have limited power and cannot reliably distinguish publication bias from asymmetry arising from genuine clinical or methodological heterogeneity or other small-study effects.

#### 3.2.3. Subgroup Analysis

**Figure 6:**
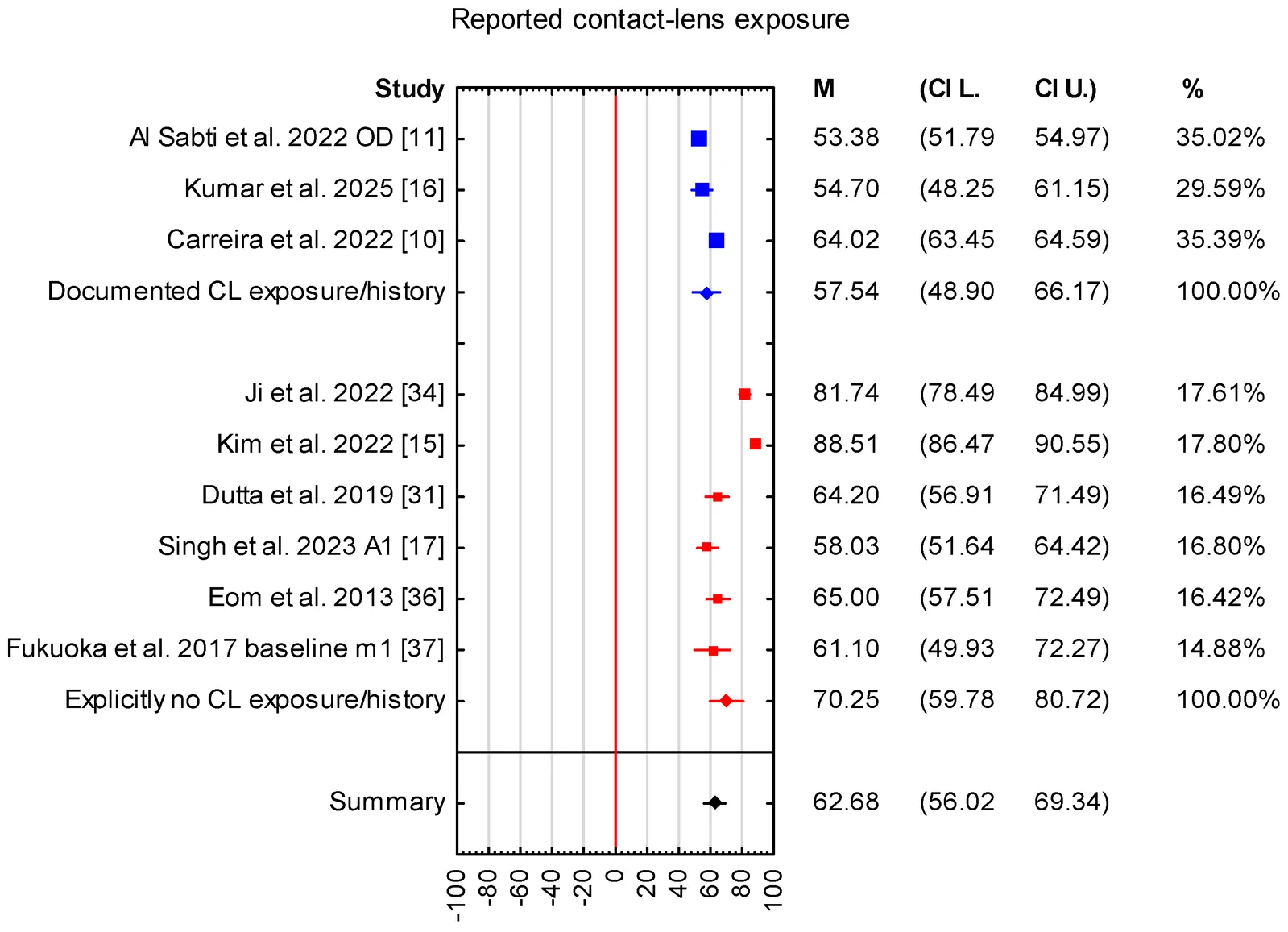
Exploratory subgroup analysis according to reported contact-lens exposure (documented exposure vs. explicitly no exposure). Blue squares represent studies with documented contact-lens exposure/history, whereas red squares represent studies with explicitly no contact-lens exposure/history. Diamonds represent pooled estimates, and horizontal lines indicate 95% CIs.

**Figure 7:**
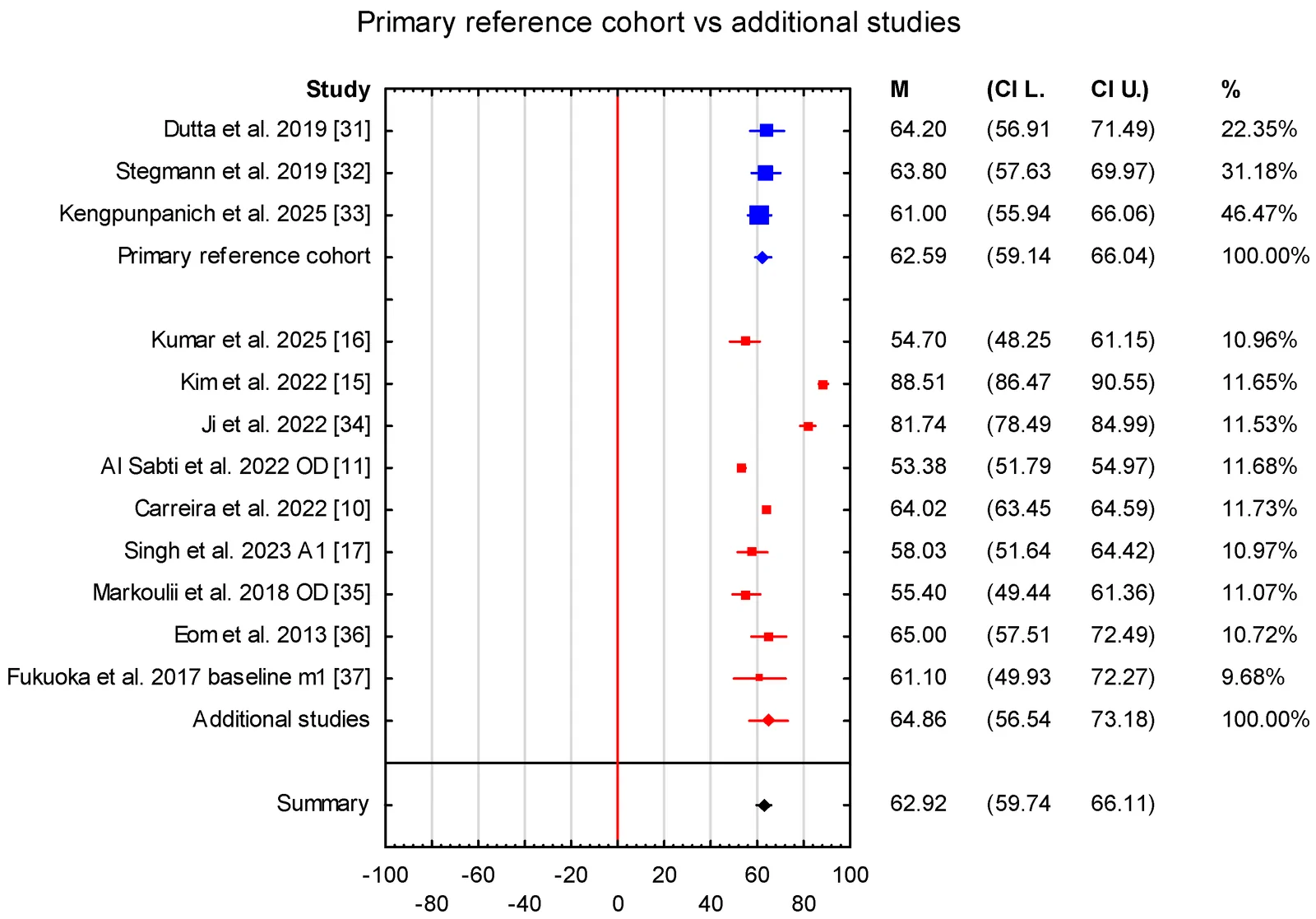
Exploratory subgroup analysis comparing the primary reference cohort with the additional studies included only in the expanded analysis. Blue squares represent studies in the primary reference cohort, whereas red squares represent additional studies. Diamonds represent pooled estimates, and horizontal lines indicate 95% CIs.

**Figure 8:**
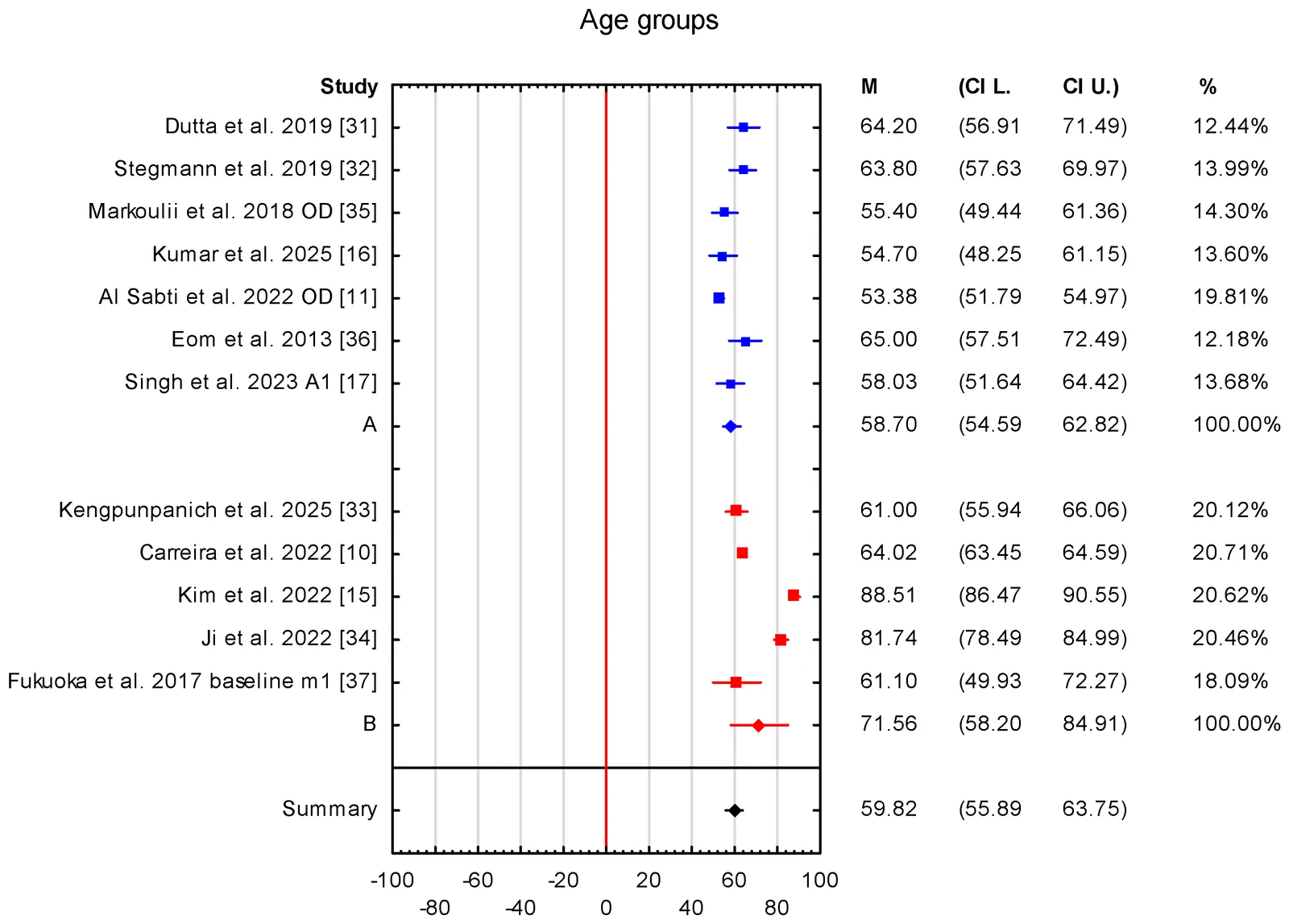
Subgroup analysis stratified by age group (A <40 years vs. B ≥40 years). Blue squares represent studies in group A (<40 years), whereas red squares represent studies in group B (≥40 years). Diamonds represent pooled estimates, and horizontal lines indicate 95% CIs.

Given the considerable heterogeneity observed in the expanded meta-analysis, exploratory subgroup analyses were conducted to investigate potential sources of between-study variability in pooled LLT estimates. The analyses were stratified according to reported contact-lens exposure (documented exposure vs. explicitly no exposure; Figure 6), population eligibility criteria (primary reference cohort vs. additional studies included only in the expanded analysis; Figure 7), and study-level age category (mean or median participant age < 40 years (A) vs. ≥40 years (B); Figure 8). For the contact-lens exposure analysis, studies reporting a history of contact-lens wear were classified as having documented exposure, irrespective of whether lens use was discontinued before LLT measurement. Studies were classified as non-exposed only when the absence of contact-lens exposure was explicitly reported; studies with unavailable, mixed, or unclear exposure information were excluded from this subgroup analysis.

In the exploratory subgroup analysis according to reported contact-lens exposure (Figure 6), studies with unavailable, unclear, or mixed exposure information were excluded. The pooled mean LLT was 57.54 nm (95% CI: 48.90–66.17 nm) across three studies with a documented history of contact-lens exposure and 70.25 nm (95% CI: 59.78–80.72 nm) across six studies explicitly reporting no contact-lens exposure. The between-subgroup difference did not reach statistical significance (Q_between = 3.37, df = 1, *p* = 0.066), although the pooled LLT was numerically lower among studies involving participants with documented contact-lens exposure. In the exploratory subgroup analysis according to population eligibility criteria (Figure 7), the pooled mean LLT was 62.59 nm (95% CI: 59.14–66.04 nm) in the primary reference cohort and 64.86 nm (95% CI: 56.54–73.18 nm) among the additional studies included only in the expanded analysis. The between-subgroup difference was not statistically significant (Q_between = 0.25, df = 1, *p* = 0.621). Study-level stratification by age (Figure 8) showed a higher pooled mean LLT among studies with a mean or median participant age of 40 years or older than among studies with a mean or median age below 40 years. The pooled mean LLT was 58.70 nm (95% CI: 54.59–62.82 nm) in Group A and 71.56 nm (95% CI: 58.20–84.91 nm) in Group B. The between-subgroup difference did not reach statistical significance (Q_between = 3.25, df = 1, *p* = 0.071), although the results suggested a possible trend toward higher LLT in studies including older participants. However, because the analysis was based on aggregate study-level age data, it should not be interpreted as evidence of an individual-level effect of age on LLT.

The subgroup analyses were repeated using left-eye estimates from Al Sabti et al. [11] and Markoulli et al. [35]. The contact-lens exposure comparison remained non-significant (57.10 vs. 70.25 nm; Q_between = 3.32, df = 1, *p* = 0.069), with a numerically lower pooled LLT among studies involving participants with documented contact-lens exposure. The population-eligibility comparison also remained non-significant (62.59 vs. 65.15 nm; Q_between = 0.30, df = 1, *p* = 0.583). Similarly, the age-group comparison remained non-significant (59.18 vs. 71.56 nm; Q_between = 2.92, df = 1, *p* = 0.088), although a numerical trend toward higher LLT in the older-study group was preserved.

## 4. Discussion

From a clinical perspective, establishing LLT reference values is highly relevant. Lipid layer thickness is increasingly used as an objective biomarker of tear film stability and meibomian gland function and is commonly incorporated into the evaluation of evaporative dry eye disease, meibomian gland dysfunction, and other ocular surface disorders [38,39]. However, meaningful interpretation of LLT measurements requires an appropriate reference framework, and published studies have reported substantial variability among apparently clinically healthy individuals. The pooled estimates presented in this meta-analysis therefore provide evidence-based reference values that may assist in the interpretation of LLT measurements in clinical practice and research settings.

Importantly, these estimates represent reference rather than normative LLT values. Although these terms are sometimes used interchangeably, they describe distinct methodological concepts. Normative values are derived from representative populations recruited specifically to characterize normal biological variation using standardized inclusion criteria and rigorous exclusion of factors known to influence the measured parameter. In contrast, reference values describe the distribution of measurements observed in clinically healthy or otherwise suitable reference populations, which may not fully represent the general population. None of the studies included in this meta-analysis was originally designed to establish population-based normative LLT values. Instead, most enrolled healthy volunteers, asymptomatic individuals, or healthy control participants selected according to study-specific eligibility criteria. Therefore, the pooled estimates should be interpreted as evidence-based reference values rather than definitive normative intervals. They may provide a study-level benchmark for contextualizing LLT values reported across populations and studies, but they cannot determine whether an individual patient’s LLT measurement is abnormally low or high.

To improve the quality and consistency of the available evidence, we applied a hierarchical study selection strategy. The primary meta-analysis included only clinically healthy or asymptomatic participants with well-characterized ocular surface status, whereas the expanded analysis additionally incorporated healthy control groups and clinically screened populations without clinically significant ocular surface disease. The similar pooled LLT estimates obtained in the primary and expanded analyses suggest that inclusion of broader clinically screened populations did not materially alter the pooled estimate. However, given differences in study designs and participant characteristics, these findings should be interpreted as supportive rather than definitive evidence of stability across populations.

Several studies reporting LLT measurements were excluded because they enrolled populations with conditions or exposures likely to affect tear film characteristics or meibomian gland function, limiting their suitability for estimating reference LLT values. These included participants with nasolacrimal duct obstruction [40], rosacea [41], thyroid eye disease [42], neurotrophic keratitis [43], or dry eye disease [44], as well as individuals undergoing upper eyelid blepharoplasty [45] or exposed to occupational or environmental factors such as prolonged face mask use [46] or rotating shift work [12]. Yoon et al. [47] were excluded despite including a nominally healthy control group because the eye selected for analysis was determined by the shorter TBUT and, when TBUT was equal between eyes, by the thinner LLT. This outcome-dependent eye-selection procedure could systematically influence the estimated mean LLT. Moreover, 6 of the 25 control participants had MGD, further limiting the suitability of this cohort as a reference population. One publication was excluded because LLT was measured in both eyes, but the study did not clearly describe how bilateral measurements were combined or otherwise reduced to a single independent participant-level LLT estimate suitable for meta-analysis [48]. Although these studies provide important information regarding LLT alterations in specific clinical or occupational contexts, they do not represent clinically healthy reference populations and were therefore not included in the present analysis.

Recent studies further emphasize that LLT is a context- and device-dependent biomarker. Ahn et al., using LipiView II for inferior corneal LLT together with complementary assessment of the superior corneal lipid layer, demonstrated that a regional LLT measurement may reflect both meibum delivery and aqueous-dependent lipid distribution [49]. Accordingly, an increased regional LLT does not necessarily indicate enhanced lipid secretion or improved tear-film function. Similarly, recent observations in symptomatic MGD indicate that LLT may increase with age despite more pronounced structural meibomian gland abnormalities, further supporting the need to interpret LLT in conjunction with age, blinking characteristics, gland morphology, and ocular surface status [50]. Moreover, Ding et al. observed longitudinal changes with a wide-field dynamic imaging system that were not reproduced by LipiView measurements, indicating that LLT results obtained with different interferometric platforms should not be considered interchangeable [51].

The subgroup analyses provided exploratory insight into potential sources of LLT variability. Studies with documented contact-lens exposure showed a numerically lower pooled mean LLT than studies explicitly reporting no contact-lens exposure. However, the between-subgroup difference did not reach statistical significance. This study-level association should therefore not be interpreted as evidence of an independent or causal effect of contact-lens exposure, particularly because the comparison was based on only three studies with documented exposure and six studies explicitly reporting no exposure and may have been influenced by differences in age, study design, device version, geographic setting, measurement protocol, and the duration or pattern of previous contact-lens use and discontinuation before LLT assessment. Although contact-lens wear could plausibly affect tear-film dynamics, TFLL stability, or meibomian gland function, the present findings cannot establish either the presence or the underlying mechanism of such an effect. The subgroup analysis based on population eligibility criteria yielded similar pooled estimates for the primary reference cohorts and the additional studies included only in the expanded analysis, with no significant between-subgroup difference, suggesting that broadening the eligibility criteria did not materially alter the overall pooled estimate. Similarly, the study-level age analysis showed a numerically higher pooled LLT among studies with a mean or median participant age of 40 years or older, but the between-subgroup difference did not reach statistical significance. Because this analysis was based on aggregate study-level age characteristics rather than individual participant data, the observed trend should be interpreted cautiously and cannot establish an individual-level association between age and LLT.

Despite these findings, the interpretation of subgroup effects should be approached cautiously. Several subgroup analyses included only a limited number of studies, which may have reduced statistical power and increased uncertainty around pooled estimates. Furthermore, the observed heterogeneity likely reflects the influence of multiple factors that could not be fully explored in the present analysis, including differences in participant selection, measurement protocols, environmental conditions, demographic characteristics, and device-specific factors. Future studies using standardized methodologies, harmonized measurement protocols, and larger study populations are needed to better identify the sources of LLT variability and establish more reliable reference values.

The stability of the pooled LLT estimate was further supported by the leave-one-out sensitivity analysis performed in the expanded meta-analysis, which demonstrated that sequential exclusion of individual studies resulted in only minor changes to the pooled estimate. This finding suggests that the overall results were not disproportionately influenced by any single study, although it does not exclude the potential contribution of methodological heterogeneity among the included datasets.

Assessment of potential publication bias and small-study effects in the expanded meta-analysis yielded inconclusive findings. Visual inspection suggested possible funnel-plot asymmetry, and the trim-and-fill procedure imputed three potentially missing studies on the right side of the plot, increasing the pooled mean LLT from 64.41 to 70.06 nm. Although the confidence intervals of the original and adjusted estimates overlapped substantially, the trim-and-fill result should not be interpreted as confirmation of publication bias. Neither Egger’s regression test nor the Begg–Mazumdar rank correlation test provided statistically significant evidence of funnel-plot asymmetry. However, given the relatively small number of included studies (*n* = 12) and the considerable between-study heterogeneity, these methods have limited power and cannot reliably distinguish selective publication from asymmetry arising from genuine clinical or methodological differences between studies or other small-study effects. Consequently, publication bias can neither be confirmed nor excluded, and these exploratory findings should be interpreted cautiously.

Several limitations of this meta-analysis should be acknowledged. First, the included studies were not originally designed to establish reference LLT values, and definitions of healthy participants, eligibility criteria, and ocular surface assessment protocols varied across studies. Second, although all LLT measurements were obtained using the LipiView ocular surface interferometer, differences in measurement procedures, participant characteristics, and environmental conditions during examination may have contributed to the observed heterogeneity. Third, potentially important determinants of LLT, including blink characteristics, ambient temperature and humidity, contact-lens wear, systemic medication use, and lifestyle-related factors, were not consistently reported, limiting the ability to perform detailed subgroup analyses or adjustments for these variables. Additionally, differences in eye-level data handling among studies (analysis of one eye, both eyes, or averaged bilateral measurements) may have influenced variance estimates. Finally, the available evidence was derived predominantly from single-center studies with limited geographic and ethnic diversity, which may restrict the generalizability of the pooled estimates. A further limitation concerns the literature search strategy. Although the updated search incorporated broader terminology related to LLT and ocular surface interferometry, it was limited to PubMed, Scopus, and Web of Science and to articles published in English. Therefore, the possibility that some eligible studies were not retrieved cannot be entirely excluded. Moreover, the review was not prospectively registered and no formal review protocol was prepared, limiting the ability to prospectively document all analytical decisions made during the review process.

Accordingly, the pooled estimates presented here should not be interpreted as population-based normative intervals, but rather as the best currently available evidence for reference LLT values measured with the LipiView ocular surface interferometer. Despite these limitations, the consistency of estimates across the primary and expanded analyses suggests that the proposed reference values may provide a useful framework for interpreting LLT measurements. Future multicenter studies involving representative population samples and standardized ocular surface assessment protocols, together with internationally accepted procedures for establishing reference intervals, are needed to determine true normative LLT values across different age groups, sexes, ethnicities, and environmental conditions.

## 5. Conclusions

This meta-analysis provides a clinically relevant, device-specific reference estimate of tear film lipid layer thickness (LLT) measured by LipiView interferometry in healthy adults and clinically screened adults without clinically significant ocular surface disease. The primary and expanded analyses yielded comparable pooled mean LLT values of approximately 62–64 nm, suggesting that the overall estimate was not markedly affected by the broader eligibility criteria applied in the expanded analysis. Although substantial heterogeneity was observed, the leave-one-out sensitivity analysis of the expanded dataset indicated that the pooled result was not disproportionately influenced by any individual study. However, the exploratory assessment of publication bias and small-study effects remained inconclusive because of the limited number of available studies and substantial between-study heterogeneity.

In clinical practice, the pooled estimate may serve as a contextual benchmark for interpreting LipiView-derived LLT measurements, but it should not be treated as a population-based normative interval, a diagnostic threshold, or a substitute for a comprehensive ocular surface assessment. Future research should prioritize prospective multicenter studies using representative sampling, standardized eligibility criteria, harmonized LipiView measurement protocols, and consistent reporting of factors that may influence TFLL, including age, sex, contact-lens exposure, blink characteristics, environmental conditions, and ocular surface status. Population-based studies are required to establish normative intervals, whereas condition-specific diagnostic-accuracy studies employing prespecified clinical reference standards are needed to determine whether LLT thresholds provide clinically useful discrimination for particular ocular surface disorders.

## Figures and Tables

**Table 1:** Risk-of-bias assessment of the included studies.

Study	Overall Risk of Bias
Dutta et al., 2019 [31]	Low-to-moderate
Stegmann et al., 2019 [32]	Low-to-moderate
Kengpunpanich et al., 2025 [33]	Low
Kumar et al., 2025 [16]	Low-to-moderate
Kim et al., 2022 [15]	Low
Ji et al., 2022 [34]	Low
Al Sabti et al., 2022 [11]	Low
Carreira et al., 2022 [10]	Low
Singh et al., 2023 [17]	Low-to-moderate
Markoulli et al., 2018 [35]	Low-to-moderate
Eom et al., 2013 [36]	Low-to-moderate
Fukuoka et al., 2017 [37]	Low

**Table 2:** Baseline characteristics of the included studies.

Study	Age Mean ± SD Range [yrs]	No. of Participants	Eye Selection	Reported Contact-Lens Exposure and Discontinuation Period	LLT Mean ± SD [nm]	Study Design
Dutta et al., 2019 [31]	22.9 ± 4.4 (18–35)	18 15 F 3 M	36 (both)	no	64.2 ± 15.8	prospective, randomized, controlled clinical study
Stegmann et al., 2019 [32]	31 ± 10	10 5 F 5 M	one eye	n/a	63.8 ± 9.96	prospective, observational, cross-sectional study
Kengpunpanich et al., 2025 [33]	total: 44.6 ± 8.5 g1 31–40 yrs: 36 ± 2.6 g2 41–50 yrs: 43 ± 2.2 g3 51–60 yrs: 55 ± 3.6	60 g1 13 F 7 M g2 12 F 8 M g3 13 F 7 M	total: 120 (both)	no contact-lens wear for 1 month before the study	total: 61 ± 20 g1 31–40 yrs: 63 ± 22 g2 41–50 yrs: 62 ± 17 g3 51–60 yrs: 57 ± 19	cross-sectional study
Kumar et al., 2025 [16]	25.4 ± 6.1 years (18–37)	20 16 F 4 M	one eye (random)	CL wearers who used CLs at least 5 days a week for 6 h per day, asked to cease lens wear for 24 h	average: 54.7 ± 14.7	prospective, randomized, bilateral, crossover trial
Kim et al., 2022 [15]	53.79 ± 8.75	30 30 F	one eye	no	88.51 ± 5.72	retrospective
Ji et al., 2022 [34]	51.86 ± 17.46 (22–78)	69 48 F 21 M	one eye	no	81.74 ± 13.79	prospective observational, cross-sectional study
Singh et al., 2023 [17]	31 ± 7.9	30 18 F 12 M	60 (both)	no	observer A, I 59.70 ± 17.21 observer A, II 58.03 ± 17.85 ^†^ observer B, I 56.70 ± 16.47	prospective
Al. Sabti et al., 2022 [11]	27 ± 6.4 (21–42)	80 48 F 32 M	160 (both)	contact-lens wear discontinued for 7 days before baseline assessment	OD baseline: 53.38 ± 7.24 OS baseline: 52.21 ± 6.95	prospective, interventional, and non-case–control study
Carreira et al., 2022 [10]	40.03 ± 6.78	42 32 F 10 M	one eye per participant	contact-lens wear discontinued for 3 months prior to enrollment	64.02 ± 1.87	prospective
Markoulli et al., 2018 [35]	20.7 ± 1.7	20 14 F 6 M	40 (both)	non-contact lens wearers or participants who had discontinued contact-lens wear for at least 24 h before each visit	OD: 55.4 ± 13.6 OS: 59.2 ± 15.5	double-masked, crossover study
Eom et al., 2013 [36]	30.4 ± 6.9	25 17 F 8 M	one eye per participant	contact-lens users were excluded	65.0 ± 19.1	cross-sectional controlled study
Fukuoka & Arita, 2017 [37]	42.4 ± 10.0	47 47 M	94 (both) ^‡^	contact-lens users were excluded	baseline: diquafosol m1 61.1 ± 39.1 saline 63.3 ± 33.6 intervention day: diquafosol 62.3 ± 31.1 saline 59.7 ± 27.4	prospective, single-center, masked trial

^†^ Selected for the meta-analysis. ^‡^ One eye was assigned to the first group (diquafosol), while the contralateral eye was assigned to the second group (saline).

## Data Availability

All articles included in the meta-analysis are accessible through the databases of their respective publishers. All results derived from the meta-analysis are explicitly reported in the article. The extracted study-level dataset and analytical selections are provided in Supplementary Table S3.

## Supplementary Materials

The following supporting information can be downloaded at https://www.mdpi.com/article/10.3390/jcm15176826/s1. PRISMA 2020 Checklist, Table S1a,b: The original and additional original search strategies, Table S2: Risk-of-bias assessment, Table S3: Extracted study-level data, Figure S1: Forest plot of the mean LLT in the expanded participant group.

## Abbreviations

The following abbreviations are used in this manuscript:

CI	confidence interval
CL	contact lens
DED	dry eye disease
GEE	generalized estimating equations
LLT	tear film lipid layer thickness
MGD	meibomian gland dysfunction
OD	oculi dextri (right eye)
OS	oculi sinistri (left eye)
SD	standard deviation
TFLL	tear film lipid layer
