# Supplementary material for: Reference Estimates of Mean Tear Film Lipid Layer Thickness Measured with LipiView® and LipiView II® in Healthy Adults: A Systematic Review and Meta-Analysis
Source: J Clin Med. 2026 Sep 3;15(17):6826. doi: 10.3390/jcm15176826 (PMC13566262; doi:10.3390/jcm15176826)
Supplement: Supplementary file 1 [file jcm-15-06826-s001.zip › jcm-4481871-supplementary.pdf]

Table S1a. Initial original search strategy designed to identify studies reporting lipid layer thickness measured using LipiView ocular surface interferometry.

Database	Search strategy	Search date
PubMed	("lipid layer thickness"[tiab]) AND (LipiView[tiab]) AND (eye[tiab])	23 August 2026
Scopus	TITLE-ABS-KEY("lipid layer thickness") AND TITLE-ABS-KEY(LipiView) AND TITLE-ABS-KEY(eye)	23 August 2026
Web of Science	TS=("lipid layer thickness") AND TS=(LipiView) AND TS=(eye)	23 August 2026

Table S1b. Additional original search strategy designed to identify studies reporting lipid layer thickness measured using LipiView ocular surface interferometry.

Database	Search strategy	Search date
PubMed	("lipid layer thickness"[tiab] OR LLT[tiab] OR "tear film lipid layer"[tiab]) AND ("LipiView"[tiab] OR "LipiView II"[tiab] OR interferometry[tiab] OR "ocular surface interferometer"[tiab])	23 August 2026
Scopus	TITLE-ABS-KEY("lipid layer thickness" OR LLT OR "tear film lipid layer") AND TITLE-ABS-KEY("LipiView" OR "LipiView II" OR interferometry OR "ocular surface interferometer")	23 August 2026
Web of Science	TS=("lipid layer thickness" OR LLT OR "tear film lipid layer") AND TS=("LipiView" OR "LipiView II" OR interferometry OR "ocular surface interferometer")	23 August 2026

Table S2. Risk-of-bias assessment on the included studies.

Study	Source design and LLT estimate	Adapted JBI Tool	Q 1	Q 2	Q 3	Q 4	Q 5	Q 6	Q 7	Q 8	Overall judgment
Dutta et al., 2019 [31]	prospective randomized controlled study; healthy baseline LLT	analytical cross-sectional	Y	U	Y	U	Y	Y	Y	Y	low-to-moderate
Stegmann et al., 2019 [32]	prospective repeated-measures observational study	analytical cross-sectional	U	U	NA	Y	U	U	U	Y	low-to-moderate
Kengpunpanich et al., 2025 [33]	cross-sectional study	analytical cross-sectional	Y	Y	Y	Y	Y	Y	Y	Y	low
Kumar et al., 2025 [16]	prospective randomized crossover study; baseline LLT	analytical cross-sectional	Y	U	Y	U	Y	Y	Y	Y	low-to-moderate
Kim et al., 2022 [15]	retrospective cross-sectional chart review	analytical cross-sectional	Y	Y	Y	Y	Y	Y	Y	Y	low
Ji et al., 2022 [34]	prospective cross-sectional study	analytical cross-sectional	Y	Y	Y	Y	Y	Y	Y	Y	low
Al Sabti et al., 2022 [11]	prospective interventional cohort; preoperative LLT	analytical cross-sectional	Y	Y	Y	Y	Y	Y	Y	Y	low
Carreira et al., 2022 [10]	prospective controlled study; healthy-control LLT	analytical cross-sectional	Y	Y	Y	Y	Y	Y	Y	Y	low
Singh et al., 2023 [17]	prospective repeatability and reproducibility study	analytical cross-sectional	Y	Y	Y	U	Y	Y	Y	Y	low-to-moderate
Markoulli et al., 2018 [35]	double-masked randomized crossover study; baseline LLT	analytical cross-sectional	Y	Y	Y	U	Y	Y	Y	Y	low-to-moderate
Eom et al., 2013 [36]	cross-sectional comparative study; healthy-control LLT	analytical cross-sectional	Y	Y	Y	Y	U	U	Y	Y	low-to-moderate

Study	Source design and LLT estimate	Adapted JBI Tool	Q1	Q2	Q3	Q4	Q5	Q6	Q7	Q8	Overall judgment
Fukuoka et al., 2017 [37]	prospective randomized contralateral-eye trial; baseline LLT in healthy participants	analytical cross-sectional	Y	Y	Y	Y	Y	Y	Y	Y	low

Abbreviations: Y, yes; U, unclear; NA, not applicable; JBI, Joanna Briggs Institute; LLT, lipid layer thickness.

Domains

Q1: Were the criteria for inclusion in the sample clearly defined?

Q2: Were objective, standard criteria used to define the study population or condition?

Q3: Was the relevant exposure or population classification measured validly and reliably?

Q4: Was LLT measured in a valid and reliable manner?

Q5: Were relevant confounding factors identified?

Q6: Were strategies for addressing confounding factors reported?

Q7: Was the statistical analysis appropriate?

Q8: Were the participants and study setting described adequately?

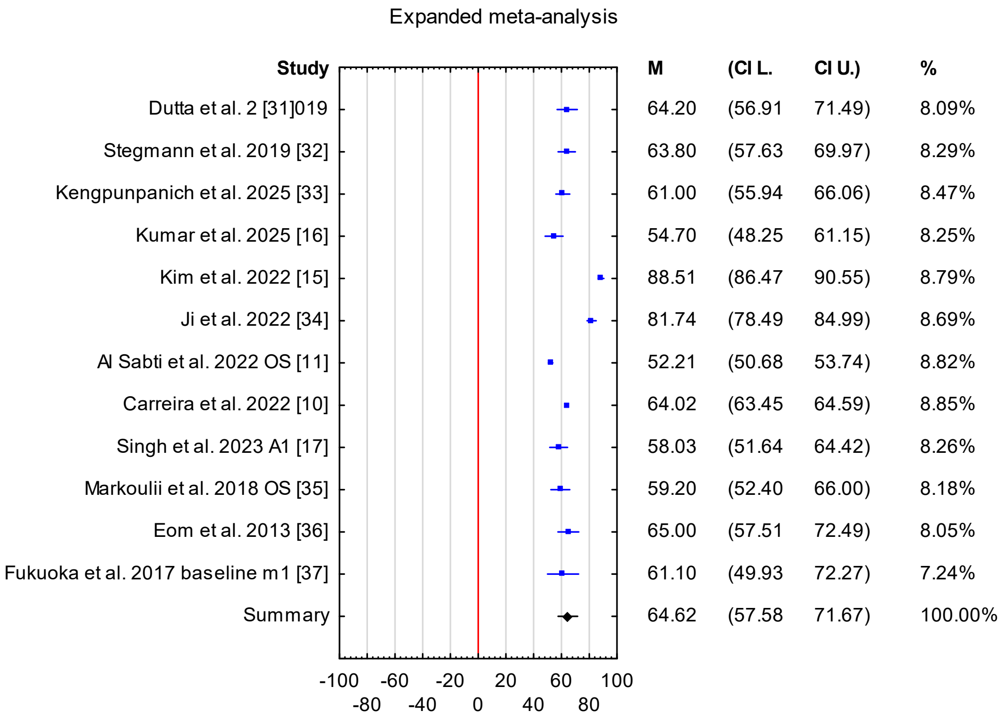

Figure S1. Forest plot of the mean LLT in the expanded participant group. The size of each box represents the point estimate for an individual study and is proportional to that study's weight in the pooled estimate. Horizontal lines indicate the 95% confidence intervals (95% CIs). OS denotes datasets in which measurements from the left eye were used in the meta-analysis.

Table S3. Extracted study-level data, selected estimates, and subgroup classifications used in the meta-analysis of tear film lipid layer thickness.

Study	n	Mean LLT (nm)	SD (nm)	SE (nm)	Population dataset	Estimate used in main analysis	CL subgroup	Age subgroup
Dutta et al., 2019 [31]	18	64.2	15.8	3.72	primary	participant-level mean of bilateral measurements	non-exposed	A
Stegmann et al., 2019 [32]	10	63.8	9.96	3.15	primary	participant-level mean from one eye per participant	n/a	A
Kengpunpanich et al., 2025 [33]	60	61	20	2.58	primary	eye-level mean of bilateral measurements; inter-eye correlation accounted for using GEE	exposed	B
Kumar et al., 2025 [16]	20	54.7	14.7	3.29	expanded only	participant-level mean of three repeated measurements from one randomly selected eye	exposed	A
Kim et al., 2022 [15]	30	88.51	5.72	1.04	expanded only	participant-level mean from one eye per participant; eye-selection method not reported	non-exposed	B
Ji et al., 2022 [34]	69	81.74	13.79	1.66	expanded only	participant-level mean from one study eye per participant; the more symptomatic eye was selected	non-exposed	B

Study	n	Mean LLT (nm)	SD (nm)	SE (nm)	Population dataset	Estimate used in main analysis	CL subgroup	Age subgroup
Singh et al., 2023 [17]	30	58.03	17.85	3.26	expanded only	eye-level mean of bilateral measurements from the second assessment by observer A	non-exposed	A
Al Sabti et al., 2022 [11]	80	53.38	7.24	0.81	expanded only	participant-level mean from the right eye at baseline	exposed	A
Carreira et al., 2022 [10]	42	64.02	1.87	0.29	expanded only	participant-level mean from one eye per participant	exposed	B
Markoulli et al., 2018 [35]	20	55.4	13.6	3.04	expanded only	participant-level mean from the right eye at the pre-instillation baseline	exposed	A
Eom et al., 2013 [36]	25	65.0	19.1	3.82	expanded only	participant-level mean from one eye per participant	non-exposed	A
Fukuoka et al., 2017 [37]	47	61	39.1	5.7	expanded only	participant-level mean from the eye randomly assigned to the diquafosol group at baseline	non-exposed	B

LLT, lipid layer thickness; CL, contact lens; SD, standard deviation; SE, standard error. The "Estimate used in main analysis" column specifies the analytical decision regarding eye, measurement, treatment group, or observer selection. The value of n represents the number of participants contributing to the selected estimate, unless otherwise indicated. SE was calculated as $\frac{SD}{\sqrt{n}}$. Primary studies were included in both the primary and expanded meta-analyses; "expanded only" indicates studies included only in the expanded analysis. CL subgroup classifications were based on reported exposure, with unclear or mixed cases excluded. Age groups were defined as <40 years (A) and ≥40 years (B) based on study-level mean or median age.
